# Presumed Pituitary Apoplexy Resulting in the Spontaneous Resolution of a Pituitary Neuroendocrine Tumor: A Case Report

**DOI:** 10.7759/cureus.61259

**Published:** 2024-05-28

**Authors:** Jordyn Mullins, Mark Bryniarski

**Affiliations:** 1 Department of Neuroscience, Burrell College of Osteopathic Medicine, Las Cruces, USA

**Keywords:** migraines, hemorrhage, adenoma, apoplexy, neuroendocrine tumor, pituitary

## Abstract

Pituitary apoplexy is a result of rapid enlargement of the pituitary, due to episodes of hyperplasia, which outpaces vascular development resulting in ischemia and potential infarction of pituitary tissue. This can present in several different ways from asymptomatic to hormonal deficiencies. Here we present a case of spontaneous reduction of a non-functioning pituitary mass, likely due to apoplexy, in which the mass went from compromising the optic chiasm to complete reduction and relief of the optic chiasm. The infarction happened spontaneously without treatment and complications. This may encourage future conservative management of pituitary tumors, rather than immediate surgical intervention.

## Introduction

Pituitary adenoma, or pituitary neuroendocrine tumor (PitNET), was first described by Pierre Marie in 1886 in relation to acromegaly [1]. Initially, it was thought that pituitary enlargement found on autopsy was a result of acromegaly. Marie was the first to discuss the notion that pituitary enlargement was rather the cause of the acromegaly. Over time, it has become well-known that PitNETs are a clonal neoplastic expansion of one or multiple cellular lineages of the pituitary gland [2]. Diagnosis is made according to the established WHO 2020 classification of tumors criteria which requires (a) sellar or suprasellar location, (b) histological features of low-grade neuroendocrine tumor that displays destruction of the normal anterior gland acinar structure, and (c) subclassification based on immunoreactivity for pituitary hormones and/or lineage-specific transcription factors [2].

The most common PitNET is non-functional (43%), followed by prolactin-secreting tumors because of lactotroph hyperplasia (40%), and the third most common being growth hormone dictates the signs and symptoms observed as a consequence of excess hormone production. A more detailed description of the physiologic activities and pathologic classification of the pituitary gland is more appropriately described elsewhere [3]. A non-functional PitNET results in no excess hormone secretion, and therefore commonly lacks systemic signs and symptoms seen with respect to functional tumors. However, there may be pituitary stalk compression, resulting in hyperprolactinemia and deficiencies in other pituitary hormones [3]. Further, it is possible that these tumors become invasive, which is more often seen in non-functional PitNETs, but this is exceptionally rare [2].

PitNETs may be further classified based on the size of the tumor; a microadenoma (< 10 mm) or macroadenoma (> 10 mm). Microadenomas present with a higher likelihood of functionality [4]. Meanwhile, macroadenomas are associated with mass effects including but not limited to headaches, photophobia, and bitemporal hemianopsia.

Pituitary apoplexy is a phenomenon that is observed when a PitNET undergoes sudden expansion resulting in hemorrhage, necrosis, and/or infarction that leads to neurologic and/or endocrinologic deterioration [4]. Often these patients will present with but not limited to abrupt headaches, visual disturbances, adrenal insufficiency, reduced mental status, cavernous sinus compression symptoms, nausea/vomiting, meningismus, and photophobia. There may also be hypothalamic involvement and suprasellar expansion that may produce acute hydrocephalus [2]. In some cases, this may be a medical emergency, and therefore needs immediate attention. If there is hemodynamic instability, altered mental status, or visual deficits it is necessary to administer intravenous (IV) corticosteroids. Surgical intervention is rarely required but may be necessary in cases where rapid decompression is required [2]. This phenomenon, if treated accordingly, is rarely fatal [5].

## Case presentation

In this case, we present a 41-year-old male with a 10-year history of migraine headaches. In the past three years, this has been accompanied by photophobia, dizziness, confusion, and memory deficits. His past medical history is significant for a sellar, cystic mass thought to be a non-functional PitNET. He also is diagnosed with hypertension, hyperlipidemia, obstructive sleep apnea, and cervical stenosis. His past surgical history is significant for C4/C5 discectomy with artificial disk replacement. The patient denied sexual dysfunction, enlargement of hands and feet, hyperhidrosis, nipple discharge, and weight changes.

**Figure 1:**
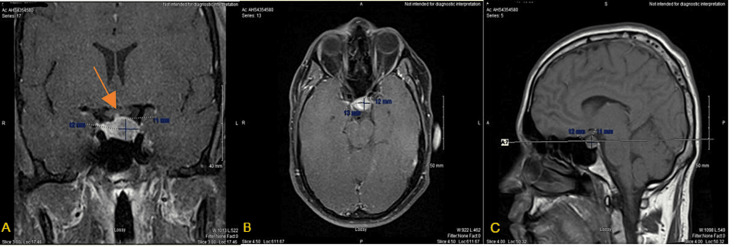
Initial MRI of a sellar mass (A) Axial T1, (B) coronal T2, and (C) sagittal T1 displaying sellar mass measuring 12 x 13 x 11 mm (L x W x H). You can observe a slight visualization of the optic chiasm (arrow in (A)).

**Figure 2:**
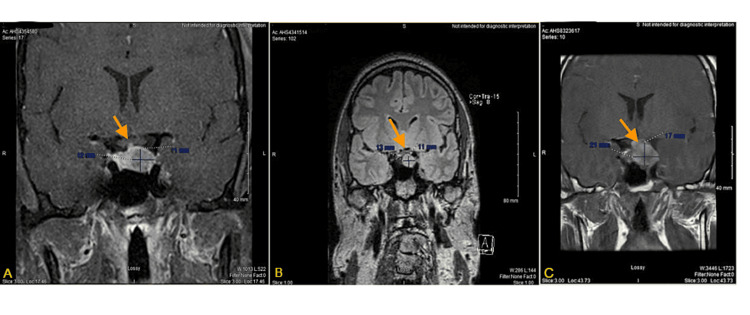
Progression of the tumor over three years from initial diagnosis: 12 x 11 mm (A) to six months after diagnosis: 13 x 11 mm (B) to just following presumed apoplexy event: 21 x 17 mm (C). The optic chiasm became compressed in second follow-up MRI (arrow in B).

The patient first presented to neurosurgery at 38 years old with episodic headaches, for which he had been treated by his primary care physician for 10 years. He began experiencing dizziness, confusion, fatigue, and pressure behind his left eye three years ago. He underwent a magnetic resonance imaging (MRI) study, which revealed a sellar mass (Figure 1). Endocrine studies were ordered which revealed physiologic levels of all pituitary hormones (Table 1). Due to a lack of optic pathway dysfunction and no history of growth, it was determined that serial examination and close follow-up were the most appropriate course of management. Follow-up with the patient was then made on a six-month basis, at which point an MRI, physical exam, and endocrine panels were repeated. His symptoms progressed with each visit, which was thought to be associated with the progression of the tumor size (Figure 2). At this time, trans-sphenoidal resection was discussed with the patient as a potential treatment to attempt to alleviate his symptoms. His endocrine panels continued to reflect physiologic hormone levels, confirming a non-functional pituitary mass (Table 1). The patient continued to be treated by his primary care physician and neurologist for migraines, which did not resolve his symptoms. Between the fourth and fifth MRI, the patient went to the emergency room with headaches that were more severe than usual, as well as mild neck stiffness.

**Table 1:** Pituitary hormone levels that displaying post-diagnosis levels at initial diagnosis (serum level 1) and levels just prior to suspected apoplexy (serum level 2). ACTH: adrenocorcotropic hormone; IGF-1: insulin-like growth factor-1; TSH: thyroid stimulating hormone; LH: luteinizing hormone; FSH: follicle simulating hormone

Hormone	Serum Level 1	Serum Level 2	Reference Range
ACTH	34	34	7-63 pg/mL
IFG-1	176	143	99-229 pg/mL
TSH	0.9	2.5	0.27-4.20 uIU/mL
Prolactin	7.3	7.5	4.0-15.2 ng/mL
LH	5.5	4.3	1.5-9.3 mIU/mL
FSH	4.5	5.7	1.4-18.1 mIU/mL

**Figure 3:**
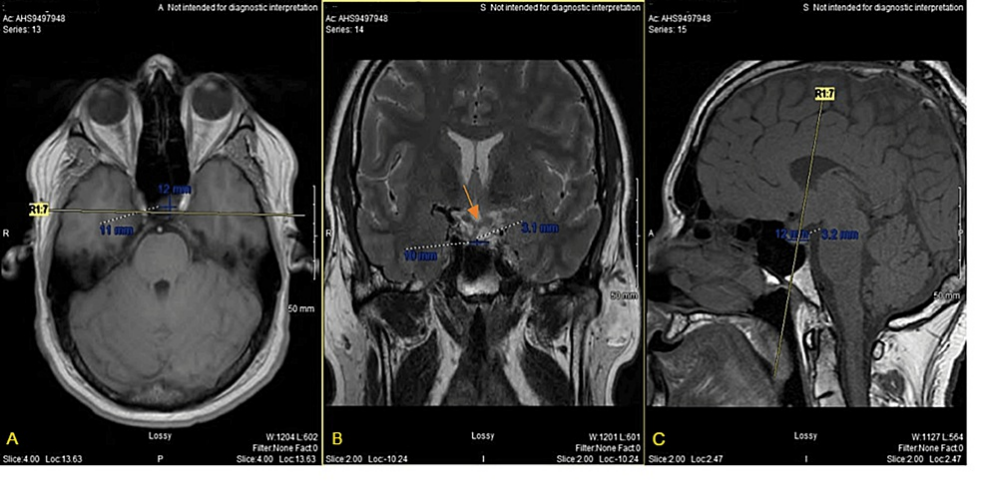
Follow-up MRI displaying near complete regression of sellar mass. (A) Axial T1, (B) coronal T2, and (C) sagittal T1 views of tumor measuring 12 x 10 x 3.1 mm (L x W x H) in the most recent MRI. This displays regression of the tumor with complete visualization of the optic chiasm (arrow in B).

Most recently he returned for a follow-up visit following an updated MRI (Figure 3). The previous four MRIs had displayed progressive enlargement of the PitNET, with eventual compression on the optic chiasm resulting in intermittent blurry vision (Figure 2, middle). The fifth, most recent, MRI displayed near complete regression of the tumor, with complete visualization of the optic chiasm (Figure 3). Following the MRI, he underwent a visual field study that showed complete visual acuity in the right eye and a very mild superonasal defect in the left eye. However, although the tumor regressed in size, the patient retained some of his symptoms present prior to tumor resolution.

## Discussion

Pituitary hemorrhage was first described by Pierce Bailey in 1898. However, the first documented use of the term apoplexy in relation to hemorrhage and necrosis of the pituitary due to rapid growth that outpaces angiogenesis was described in 1950 [6]. Apoplexy may occur with PitNETs that rapidly increase in size, or under other similar etiologies that lead to strokes, such as embolization of an atherosclerotic plaque [7,8]. This phenomenon is often associated with symptoms including acute headaches, visual deficits, meningismus, and hormonal abnormalities [2].

While pituitary apoplexy may be an emergent condition, when treated appropriately mortality is drastically reduced by around 1.6% [7]. Controversy exists with respect to the management of these patients, with some believing that trans-sphenoidal decompression is necessary immediately. Meanwhile, other physicians prefer to reserve this approach for scenarios where deterioration of consciousness, vision, and hypothalamic functions are present. In the absence of this, it is recommended to treat with fluids, for hemodynamic stability, as well as corticosteroids, to avoid an adrenal crisis [7]. To our knowledge spontaneous apoplexy leading to resolution of such tumors has been reported in 12 other manuscripts following a brief search through PubMed [9]. This report aims to encourage the discussion between immediate surgical intervention and conservative management of PitNETs.

**Figure 4:**
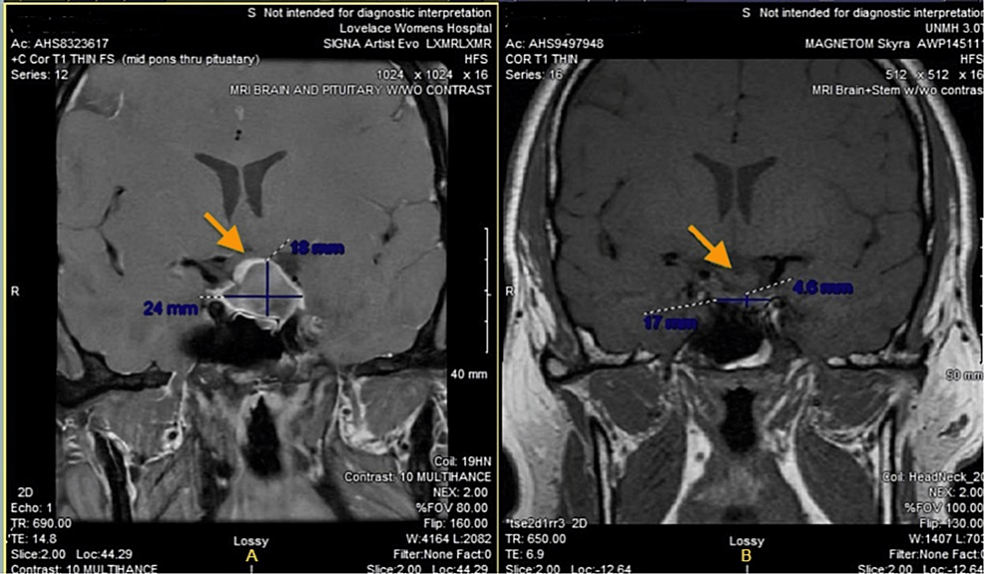
(A) Enlargement of the pituitary mass showing compression of the optic chiasm (orange arrow) (24 x 18 mm). (B) Visualization of the optic chiasm with a drastic reduction in the size of PitNET alleviating the optic chiasm of mass effect (orange arrow) (17 x 4.6 mm). PitNET: pituitary neuroendocrine tumor

The patient reported in this case most likely underwent pituitary apoplexy when he presented to the emergency department with unusually intense headaches, pain from his left ear to his jaw, meningismus, and visual abnormalities. The patient refused treatment and was discharged from the hospital. The next day he underwent an MRI that showed an increase in the size of the tumor, compared to the previous MRI (Figure 4A). The patient continued treatment and was seen again for a follow-up six months later. This MRI showed near complete regression, leading to the postulation that apoplexy likely occurred (Figure 4B). The necrosis of the mass naturally led to decompression of adjacent tissue structures, most notably the optic chiasm (Figure 4B). It is worth noting that the result of the spontaneous event resulted in decompression of adjacent structures that would have been adequate if surgical resection had been attempted. Through decompression, it would be expected that some, or all, of the symptoms would regress. However, this patient’s symptoms did not, leaving no clear etiology to explain his headaches, photophobia, dizziness, and confusion that continue to persist. There was discussion that potentially, the tissue was impeding the cavernous sinus. This compression of the sinus may potentially irritate the trigeminal nerve, leading to symptoms of migraines.

## Conclusions

It is worth reporting cases where pituitary apoplexy may lead to spontaneous resolution of the tumor mass in a similar way to surgery, which could lead to the resolution of symptoms. This may be another leg on which to stand when defending conservative measures toward the management of PitNETs and pituitary apoplexy versus emergent decompression through a surgical approach.

We will continue to follow up with this patient to monitor any anatomic changes to the tumor. It will be important to ensure that pituitary function remains intact, as it is more common for non-operative cases to result in hypopituitarism. He will continue to see neurology and physical medicine and rehabilitation to manage his headaches, photophobia, dizziness, and confusion. The postulation that symptom maintenance may have been due to cavernous sinus irritation will require follow-up. The aim would be that resolution is obtained over a period of time, assuming there is no return of the tumor. Our role, as neurosurgery, will be accomplished through follow-up MRI of the brain every six months to ensure that the tumor does not return. If there is a recurrence of the tumor, it will be important to again revisit the behavior of the tumor through pituitary hormone panels and frequent imaging. If necessary, surgical intervention may be appropriate to establish tumor type as well as therapeutic resolution.

## References

[1] de Herder WW (2009). Acromegaly and gigantism in the medical literature. Case descriptions in the era before and the early years after the initial publication of Pierre Marie (1886). Pituitary.

[2] Greenberg MS (2023). Greenberg’s Handbook of Neurosurgery. Tenth edition.

[3] Kumar V, Abbas AK, Aster JC, Perkins JA (2018). Robbins Basic Pathology. Tenth Edition.

[4] Russ S, Anastasopoulou C, Shafiq I (2023). Pituitary adenoma. StatPearls [Internet].

[5] Singh TD, Valizadeh N, Meyer FB, Atkinson JL, Erickson D, Rabinstein AA (2015). Management and outcomes of pituitary apoplexy. J Neurosurg.

[6] Brougham M, Heusner AP, Adams RD (1950). Acute degenerative changes in adenomas of the pituitary body - with special reference to pituitary apoplexy. J Neurosurg.

[7] Mayol Del Valle M, De Jesus O (2023). Pituitary apoplexy. StatPearls [Internet].

[8] Nawar RN, AbdelMannan D, Selman WR, Arafah BM (2008). Pituitary tumor apoplexy: a review. J Intensive Care Med.

[9] Eichberg DG, Di L, Shah AH, Kaye WA, Komotar RJ (2020). Spontaneous preoperative pituitary adenoma resolution following apoplexy: a case presentation and literature review. Br J Neurosurg.

